# The Efficacy of Psycho-Educational Group Program on Medication Adherence and Global Functioning of Patients with Bipolar Disorder Type I

**Published:** 2014-01

**Authors:** Mohammad Jafar Bahredar, Ali Asghar Asgharnejad Farid, Ahmad Ghanizadeh, Behrooz Birashk

**Affiliations:** 1 Department of Clinical Psychology, Tehran Institue of Psychiatry, Iran University of Medical Science, Tehran, Iran;; 2 Mental Health Research Center, Department of Clinical Psychology, Tehran Institue of Psychiatry ,Iran University of Medical Science, Tehran, Iran;; 3 Research Center for Psychiatry and Behaviour Science, Department of Psychiatry, Shiraz University of Medical Sciences, Shiraz, Iran

**Keywords:** Bipolar Disorder, Psycho-Education, Medication Adherence, Function

## Abstract

**Background: **Psycho-education is now considered as part of the integrated treatment for bipolar disorder. This study aimed to determine the efficacy of group psycho-education on medication adherence and global functioning of patients with bipolar disorder type I.

**Methods:** 45 patients with bipolar disorder type I were allocated one of the three groups of psycho-education plus pharmacotherapy, pharmacotherapy and placebo plus pharmacotherapy. A psycho-educational program was conducted for the psycho-educational group during 9 weekly sessions. Medication adherence and global functioning of all the three groups were evaluated before the intervention, three months and six months after the intervention using Medication Adherence Rating Scale (MARS) and Global Assessment of Functioning (GAF). ANOVA was performed to examine the data.

**Results: **In the first and second assessments, the mean score of medication adherence and gobal functioning for patients in the psycho-educational group was significantly higher than that in the control and placebo groups (P=0.001). Medication adherence score of the psycho-educational group was increased from 6.27(0.88) to 7.92(1.38). while the mean score of the psycho-educational group increased from 56.6 (3.58) to 64.17 (2.12):, the global functioning reduced from 56.27(3.17) to 54.17(5.08) in the control group and from 56.67 (3.58) to 56 (4.36) in the placebo group.

**Conclusion:** Psycho-educational program plus pharmacotherapy was effective in improvement medication adherence and global functioning of bipolar patients.

## Introduction

Bipolar disorder is a chronic psychiatric disorder that often causes disability and performance degradation in patients and also affects the life quality of the patients and their family members.^1^ As this disorder is chronic and reversible, it is the sixth cause of disability in the world among medical and psychiatric disorders.^2^ This disorder results in social, occupational and financial problems, deterioration of marital relation and also suicide.^3^^,^^4^ The risk of committing suicide in these patients was 15 times greater than that in the general population and it seems that performance degradation of these patients will even stay in the recovery periods. 62% of bipolar patients reported that they are not satisfied with their personal lives, and many of them complained about the noncompliance with social and occupational conditions.^5^^,^^6^

One of the reasons of the development of bipolar disorder in patients is poor adherence treatment. Poor cooperative treatment is one of the main challenges to control the symptoms and prevent the relapse into bipolar disorder.^7^ Some studies noted the fact that only about 40% of the patients have the required cooperation with taking their drug regime recommended by the doctor and this involvement is even lower than that related to the patients with schizophrenia who had adherence treatment of 50 to 60 percent.^8^

Researchers considered several factors which affect poor cooperative treatment in bipolar patients. such factors as early age in which the disorder starts, committing suicide and hospitalization 12 months before the severity of the bipolar disorder, the existence of more mania and mixed states, rapid cycling periods, and symptoms of delusion and hallucination.^7^ Also factors related to treatment such as drugs’ side effects and efficacy can be effective in poor adherence treatment and some people consider it as the seventh reason for drug withdrawal by the patient.^9^ Side effects such as gaining weight, degradation of cognitive performance and intensified depression symptoms following the drug intakes are effective factors in poor adherence treatment. Low effectiveness of the prescribed medications has also been proposed as a risk factor for poor cooperative treatment.^7^ Other effective factors in poor cooperative medication are psychological factors. Among the psychological factors affecting poor cooperative medication, the insight and awareness about the disease and the patient’s attitude towards the medication can be mentioned.^10^ Previous studies pointed out that low insight can be associated with poor cooperative treatment. Therapists believe that low awareness about the disease play an important role in cooperative medication.  Patients who are not aware of their disease follow the instructions less carefully. These patients do not cooperate well in psychological treatments.^10^^,^^11^

Various studies showed that psychological treatments can have positive effects on enhancing bipolar patients’ medication adherence. One of these treatments is centralized interventions on psycho-education.^12^ This study aimed to investigate the effectiveness of the psycho-educational group program on medication adherence and global functioning of patients with bipolar disorder.

## Materials and Methods

This experimental study was done from October 2012-July 2013. The assessments occurred at, pre- and post-tests as 3 and 6 month assessments, the patients were allocated one of the three groups: a) psycho-educational group plus pharmacotherapy), b) the placebo group plus pharmacotherapy and c) pharmacotherapy alone. In this study, the independent variable is psycho-education and the dependent variables are the changes resulting from the application of the independent variables (changes in medication adherence and overall performance of the patients). This study was approved by the ethics committee of Tehran University of Medical Sciences. The patients provided written informed consent for their participation

In this study, the statistical samples included patients with bipolar disorder type I in Shiraz. The samples were selected from patients who referred to psychiatric private clinics and governmental centers of Shiraz University of Medical Sciences, Hafez and Ibn Sina hospitals. Sampling was based on purposive sampling and availability. There were several (inclusion) criteria for in this study: being diagnosed as bipolar disorder type I based on diagnostic interview and SCID-I/CV test results. Being in the age range of 18 to 50, having the euthymic mood during the study (score less than or equal to 7 for Hamilton scale and score less than or equal to 6 for Young Mania scale), having the patients’ agreement to participate in the study by signing a written informed consent, and not receiving psychological treatment simultaneously at the beginning or before entering the study. The exclusion criteria of the study were being at the acute phase of the disease or being an active drug abuser, having the full criteria for borderline personality disorder diagnosed by psychiatrist and according to SCID-II test results, having serious physical problems that hinder the person’s presence in the group, having mental retardation, being in the age range of below 18 and above 50.

After checking the inclusion and exclusion criteria, 45 patients with bipolar disorder type I were selected and categorized into three groups: the experimental group (n=15), the control group (n=15) and the placebo group (n=15). Patients in all three groups were matched with each other regarding severity of depression and mania (P=0.408 and 0.410 respectively) the number of hospitalizations and having ECT (P=0.710 and 0.757). For the experimental group, psycho-educational program were performed in nine weekly sessions according to psycho-educational program of Colom et al.^9^ The content of psycho-educational group program consisted of bipolar disorder definition and its causes, an introduction to the symptoms of mania and hypomania, an introduction to the symptoms of depression and mixed states, the process and prognosis of the disease, the mood stabilizer medications, medications for treatment of mania and depression, learning early identification of disorder, the activities patients should do at the beginning of the disorder and drug misuse. The control group continued their routine treatment including monthly regular visits with a psychiatrist and taking medication. The placebo group, in addition to drug therapy, had about 15-20 minute sessions with a clinical psychologist for 5 weeks. These sessions were just supportive and no psycho-education was given to patients. Patients in all the three groups completed the research tests three and six months after the intervention. In the fifth week, one of the patients of the control group was excluded from the study due to the relapse of the disease and hospitalization and two other patients did not attend the second and third assessment sessions due to travel. For this reason, the number of individuals in the experimental group was reduced to 12. Also, three patients of the control group did not attend the second and third assessments, so the number of individuals in this group was reduced to 12. In the placebo group, 4 patients did not attend the second and third assessment sessions due to travel and relapse of the disease. It should be noted that according to Colom et al.^9^ the appropriate number of individuals for psycho-educational program was about 8 to 12 individuals. 15 patients in each group were chosen to compensate for the loss of patients.

Medication Adherence Rating Scale (MARS) and Global Assessment of Functioning (GAF) were completed for all the three groups before the intervention, three months, and six months after the intervention. The patients also completed HAMD-24 and YAMRS before the study. Hamilton Rating Scale for Depression^13^ is an instrument used to assess the severity of depression which is completed by doctors or clinical assessments. The validity of this scale its correlation with other instruments ranged 0.60 to 0.84 and also the validity of the internal assessment was 0.84-0.90. The Young Mania Rating Scale was used to measure the severity of mania. This questionnaire contains 11 questions that were filled out by a psychiatrist and a trained person.^14^ The Cronbach’s alpha coefficient was 0.72 and the correlation coefficient between raters was 0.96.^15^

The assessment medication adherence scale was used to assess the patients’ medication adherence. This simple questionnaire consists of 10 questions with yes or no responses which can be easily answered by the patient or therapist. The test-retest reliability coefficient for this test was 0.91.^16^ GAF which is a hypothetical continuum for occupational, social and psychological performances in psychological disorders was used to evaluate the performance of the test subjects. This questionnaire shows the mental malfunctions but does not show those related to the physical or environmental restrictions.^17^

SCID-I (Structured Clinical Interview for DSM-IV Axis I disorders) is a standardized comprehensive instrument to assess major psychiatric disorders according to DSM-IV criteria which has been designed for research and clinical purposes.^18^ All of SCID-I is usually administered in a session and takes time from 45 to 90 minutes. The Persian translation of the SCID yields diagnoses with acceptable to good reliability and validity in a clinical population In Iran  diagnostic agreements between test and retest SCID administration were fair to good for most diagnostic categories. Overall weighted kappa was 0.52 for current diagnoses and 0.55 for lifetime diagnoses. Specificity values for most psychiatric disorders were high (>0.85); however the sensitivity values were somewhat lower.^19^ SCID-II as well as SCID-I is a semi-structured diagnostic interview which has been prepared to measure 10 personality disorders on Axis II and also depressed personality disorder and passive-aggressive personality disorder in NOS section of Axis II. Its implementation takes less than 20 minutes. Reliability and validity of this scale have been reported desirable.^19^^,^^20^

*Data Analysis*

For the analysis of data such as age, age of disease onset and education, one way ANOVA was used. For comparison of medication adherence and global functioning of patients in three groups, ANOVA with repeated measures were used. Data analysis was performed using SPSS 16 statistical program.

## Results

The subjects of the three groups of psycho-educational group plus pharmacotherapy, pharmacotherapy group (control) and placebo group plus pharmacotherapy were not significant in terms of major demographic variables (age, age of disease onset, and education) based on one-way ANOVA (table 1). There were 7, 9 and 8 female participants in each of these three groups, respectively. Also there were 6, 6,8 married subject in each groups respectively. The subjects of three groups were not significantly different in terms of marital status (P=0.698). About 2,4 and 4 participants in each of these three groups were employed(P=0.580)

**Table 1:** Mean and standard deviation for age, age of disease onset and education of the three groups

** Groups** **Variables**	**Psycho-education** **mean±SD**	**Control** **mean±SD**	**Placebo** **mean±SD**	**F**	**P**
Age	29.73**±**5.50	29.66**±**5.28	31.60**±**6.36	0.543	0.585
Age of disease onset	23.06**±**5.52	23**±**4.76	23.33**±**4.27	0.020	0.981
Education	10.93**±**2.31	10.80**±**2.27	11**±**2.07	0.032	0.969

*Medication Adherence*

**Figure 1:**
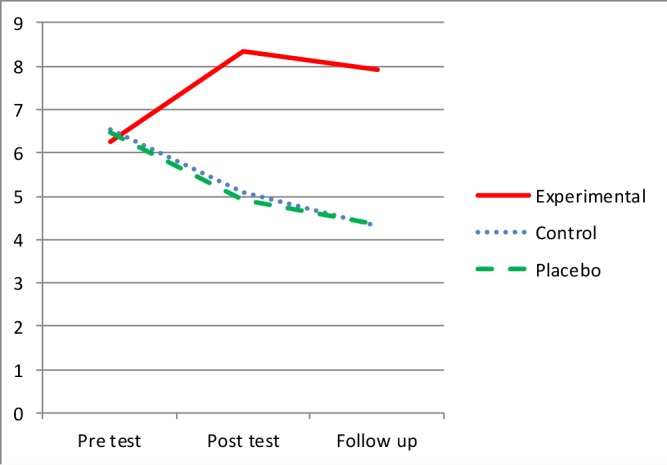
The medication Adherence in the group

Medication adherence mean score before the intervention (baseline) did not show a significant difference among the three groups (P=0.55). Medication adherence score (mean±SD) of the psycho-educational group was increased from 6.27±0.88 to 7.92±1.38 in six months after the intervention. But was reduced from 6.53±0.64 to 4.33±0.49 in the control group and from 6.47±0.52 to 4.36±0.67 in the placebo group (table 2). The differences between the groups were statistically significant [F (2, 31)=55.09, P=0.0001] (figure 1). Although the mean score of the psycho-educational group in the second assessment was a little lower than that in the first assessment, this difference was not statistically significant (P=0.72). But the medication adherence scores in the first and second assessments were statistically significant for the control and placebo groups (P=0.016) and (P=0.001). (figure 1)

**Table 2:** Mean and standard deviation of medication adherence and overall performance of the three groups

**Variables**	**Groups** **(mean**±**SD)**	**Baseline** **(mean**±**SD)**	**First assessment** **(mean**±**SD)**	**Second assessment** **(mean**±**SD)**	**Differences between the groups**
Medication adherence	Psycho-educational	6.27±0.88	8.33**±**0.65	7.92±1.38)	[F(2,31)=55.09, P=0.0001]
	Control	6.53±0.64	5.08**±**0.79	4.33±0.49)	
	Placebo	6.47±0.52	4.91±0.54	4.36±0.67)	
Global functioning	Psycho-educational	56.6±3.58	64.83**±**1.9	64.17±2.12)	[F(2,31)=90.93, P=0.0001]
	Control	56.27±3.17	55.25±3.91	54.17±5.08)	
	Placebo	56.67±4.5	56.27±3.6	56.0±4.36)	

*Global Functioning*

**Figure 2:**
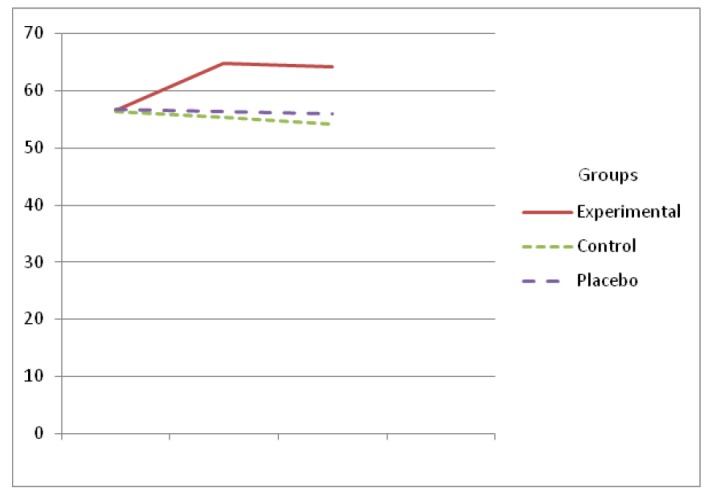
The global functioning in the group

Global functioning mean score before the intervention (baseline) did not reveal a significant difference among the three groups (P=0.944). However, the mean score of the psycho-educational group was increased from 56.6±3.58 to 64.17±2.12 in the second assessment, the global functioning was reduced from 56.27±3.17 to 54.17±5.08 in the control group and from 56.67±3.58 to 56±4.36 in the placebo group. The differences among the groups were statistically significant [F (2, 31) = 90.93 P=0.0001] (table 2). The global functioning of the psycho-educational group was significantly higher than the placebo and control groups (figure 2).

## Discussion

The main finding of this study demonstrated the effectiveness of psychological interventions based on psycho-educational model plus conventional therapies for improving medication adherence and global functioning in bipolar patients type I. In this study, medication adherence and global functioning of the bipolar patients in the first assessment (three months after the intervention) and the second assessment (six months after the intervention) were significantly higher than those of the control and placebo groups. The positive effect of psycho-education on patients’ medication adherence and global functioning has been investigated in several studies. The findings of this study were in line with some other studies. These studies showed the positive effects of psycho-educational program on medication adherence of Lithium and other mood stabilizers. These studies demonstrated that psycho-educational program can have profound effects on the disorder and its treatment in bipolar patients.^21^^-^^23^ Also, the findings of the present study are in line with of Dsouza et al.^24^ They found that participating in psycho-educational programs increases adherence treatment of patients.

Psycho-educational program can target effective psychological factors in poor adherence treatment. Studies conducted by another researcher showed that low insight in poor cooperative treatment was effective.^10^ Other studies found that the role of low insight about the disease and the patient’s attitude about the medication has been effective on poor adherence treatment. Feeling dissatisfied with life and its low quality is associated with poor cooperation in drug treatments as well.^11^ According to Colom et al.^9^ one of the mechanisms of psycho-educational program is raising awareness about the disease and the mechanism of medicines’ effects and side effects. Leclerc et al.^7^ believed that psycho-educational program can increase the patient’s awareness, reduce the number of relapses and increase the patients’ quality of life.

Another finding of this research was increase in the global functioning of patients on whom the psycho-educational program had been administered. In this study, the global functioning of the patients of the three groups was not significant in the pre-test phase,. However, the global functioning of the experimental group was significantly higher than that of the control and the placebo groups in the first and second assessments. The findings of this research were in line with other investigations on the effectiveness of psycho-educational program used to improve the global functioning of bipolar patients.^25^^,^^26^ Although several factors are effective in reducing the performance of bipolar patients, it has been revealed that self self stigma is accompanied by the most  degradation of the patients performance.^27^ In another study on 80 bipolar patients, self-stigma was negatively correlated with the global functioning of the individual.^28^ It has been found that tags caused by the psychiatric disease were significantly associated with disruption of interpersonal relationships, cognitive and leisure functions and could affect autonomous, occupational and financial spheres.^29^ Another mechanism of psycho-educational program is awareness about the causes of the disease and factors of the disease onset. Identifying the underlying causes of the disease and also warning symptoms can increase the patients’ confidence and reduce the anxiety of the patients and their families. It is believed that psycho-educational program is not only a powerful tool to increase adherence treatment, but also helps the patients to control their own concerns, fears, personal and social stigma and low self-esteem.^30^ Psycho-educational program can even be used as a mood-stabilizing agent. In the present study, the role of the patient’s awareness about the genetic and biological causes of bipolar disorder had a significant effect on adherence treatment and subsequently increased the global functioning of the patients. Limitations of this study include the small sample size and short assessment period. It is recommended that future studies apply longer time period and larger sample sizes in this area.

## Conclusion

Psycho-educational group therapy not only increases the rate of adherence to medication therapy but also increases global functioning of the patients with bipolar disorder. Group psycho-education as part of bipolar disorder management is highly recommended.
